# Prognostic value of tumor-infiltrating lymphocytes in patients with triple-negative breast cancer: a systematic review and meta-analysis

**DOI:** 10.1186/s12885-020-6668-z

**Published:** 2020-03-04

**Authors:** Guoxuan Gao, Zihan Wang, Xiang Qu, Zhongtao Zhang

**Affiliations:** Department of General Surgery, Beijing Friendship Hospital, Capital Medical University, Beijing Key Laboratory of Cancer Invasion and Metastasis Research & National Clinical Research Center for Digestive Diseases, 95 Yong-an Road, Beijing, 100050 China

**Keywords:** Triple-negative breast cancer, Tumor-infiltrating lymphocytes, Prognosis, Meta-analysis

## Abstract

**Background:**

The objective of this systematic review and meta-analysis was to determine the prognostic value of total tumor-infiltrating lymphocytes (TILs) and subtypes of TILs (CD4^+^, CD8^+^, and FOXP3^+^) in triple-negative breast cancer (TNBC).

**Methods:**

A systematic search of the MEDLINE, EMBASE, and Web of Science databases was conducted to identified eligible articles published before August 2019. Study screening, data extraction, and risk of bias assessment were performed by two independent reviewers. Risk of bias on the study level was assessed using the ROBINS I tool and Quality in Prognosis Studies (QUIPS) tool. We performed a meta-analysis to obtain a pooled estimate of the prognostic role of TILs using Review Manager 5.3.

**Results:**

In total, 37 studies were included in the final analysis. Compared to TNBC patients with low TIL levels, TNBC patients with high TIL levels showed a higher rate of pathological complete response (pCR) to treatment (odds ratio [OR] 2.14, 95% confidence interval [CI] 1.43–3.19). With each 10% increase in percentage of TILs, patients with TNBC had an increased pCR (OR 1.09, 95% CI 1.02–1.16). Compared to TNBC patients with low TIL levels, patients with high TIL levels had better overall survival (OS; hazard ratio [HR] 0.58, 95% CI 0.48–0.71) and disease-free survival (DFS; HR 0.66, 95% CI 0.57–0.76). Additionally, with a continuous increase in TIL levels, patients with TNBC had improved OS (HR 0.90, 95% CI 0.87–0.93) and DFS (HR 0.92, 95% CI 0.90–0.95). A high CD4^+^ TIL level was associated with better OS (HR 0.49, 95% CI 0.32–0.76) and DFS (HR 0.54, 95% CI 0.36–0.80). A high CD8^+^ TIL level was associated better DFS only (HR 0.55, 95% CI 0.38–0.81), as no statistical association was found with OS (HR 0.70, 95% CI 0.46–1.06). A high FOXP3^+^ TIL level also was associated with only DFS (HR 0.50, 95% CI 0.33–0.75) and not OS (HR 1.28, 95% CI 0.24–6.88).

**Conclusions:**

TNBC with a high level of TILs showed better short-term and long-term prognoses. High levels of specific phenotypes of TILs (CD4^+^, CD8^+^, and FOXP3^+^) were predictive of a positive long-term prognosis for TNBC.

## Background

Triple-negative breast cancer (TNBC) is the term used to describe breast cancer cases that lack expression of estrogen receptor (ER), human epidermal growth factor receptor-2 (HER2), and progesterone receptor (PR) [1]. TNBC is characterized by a poor prognosis, and accordingly, the 5-year survival rate is only around 60% [2]. As the malignancy of breast cancer depends not only on its genetic abnormalities and biological characteristics but also on interactions between the cancer cells and their microenvironment, it is vital to understand the tumor microenvironment [3].

The microenvironment of breast cancer contains a variety of cell types, including tumor-infiltrating lymphocytes (TILs). Accumulating evidence indicates that TILs play essential roles in carcinogenesis and cancer progression [4]. Furthermore, interleukin (IL)-6 and IL-8 secreted by some subtypes of lymphocytes can generate a positive feedback loop between the immune microenvironment and tumor cells [5]. According to the results of a meta-analysis in 2014, the level of TILs was positively associated with a the prognosis of TNBC [6]. However, various subtypes of TILs have both inhibitory and stimulatory effects on the prognosis and progression of breast cancer. The CD4^+^ T cells and CD8^+^ T cells (primary effector TIL subtypes) have been linked to a better response to systemic treatment in breast cancer [7, 8]. On the contrary, FOXP3^+^ T-cell infiltration was found to predict a worse prognosis via the mediation of tumor immune escape [9, 10]. Because TNBC has unique clinicopathological and immunohistochemical features, determining the clinical associations of the total TIL count or the levels of specific subtypes of TILs in TNBC can improve our ability to predict the prognostic pattern and treatment response for TNBC.

The objective of the present systematic review and meta-analysis was to determine the prognostic roles of the total TILs or the levels of subtypes of TILs (CD4^+^, CD8^+^, and FOXP3^+^) in TNBC.

## Methods

The present systematic review and meta-analysis were conducted following the requirements of the Preferred Reporting Items for Systematic Reviews and Meta-Analyses (PRISMA) statement [11].

### Search strategy and study selection

A systematic literature search was conducted using the MEDLINE, EMBASE, and Web of Science databases to identify eligible articles published before August 2019. The keywords used for the literature search included triple-negative breast cancer (TNBC), tumor-infiltrating lymphocytes (TILs), prognosis, and survival. Review and meta-analysis articles were scanned for additional relevant studies. The literature search strategies are outlined in Additional file 1.

### Outcome definitions

Pathological complete response (pCR) was defined as the absence of all invasive disease cells and lymph node metastasis [12]. Overall survival (OS) was defined as the period from the date of TNBC diagnosis to the time of death with any cause [13]. Disease-free survival (DFS) was defined as the period from the start of treatment to the first recurrence, or to death without any type of relapse [13].

### Inclusion and exclusion criteria

The inclusion criteria were the following: (1) paper written in English, (2) study population or study sub-group consisted of patients with TNBC, (3) the relationships between TIL levels and short-term prognosis (i.e., pCR) and long-term prognosis (i.e., OS and DFS) were investigated, (4) original studies without restriction in study design, (5) studies containing enough data to estimate the effects (i.e., hazard ratios [HRs] and corresponding 95% confidence intervals [CIs] for OS or DFS, and odds ratios [ORs] and corresponding 95% CIs for pCR). The exclusion criteria were the following: (1) reviews, commentaries, editorials, protocols, case reports, qualitative research, or letters; (2) duplicate publications; and (3) full text not published in English, and (4) studies without usable data.

### Study selection and quality assessment

Title–abstract screening was performed first to determine eligibility by two independent reviewers. Full-text articles that passed the first stage screening were downloaded for further review according to the inclusion and exclusion criteria. Disagreements were resolved by consultation with a third author or by joint discussion.

As no randomized controlled trial was found, we assessed the risk of bias using an approach based on the ROBINS I tool [14] and the Quality In Prognosis Studies (QUIPS) tool [15]. The risk of bias assessment was conducted by two reviewers independently.

### Data extraction

We extracted data from the included studies using a pilot-tested data extraction form. We extracted the following data for this review: (1) first author and publication year, (2) country in which study was conducted, (3) study design, (4) participant details, (5) duration of follow-up, (6) choice of cut-off scores for defining positive TILs, (7) TIL category, (8) TIL measurement details (category or continuous). The definition of high/low TIL level were attributed to the original papers. (9) adjusted HRs with 95% CIs for OS and/or DFS (univariable HRs were recorded only if adjusted HRs were not available), and (10) adjusted ORs with 95% CIs (or accurate event numbers) for pCR (univariable ORs were recorded only if adjusted ORs were not available).

### Statistical analysis

We performed meta-analyses to obtain a pooled estimate of the prognostic role of TILs using RevMan 5.3. Category software, and continuous TILs were estimated separately to decrease the heterogeneity. The results were expressed as HR (95%CI) for OS and DFS and by OR (95% CI) as calculated by Review Manager 5.3 [16]. A *P*-value less than 0.05 was set as indicative of statistical significance. Between-study heterogeneity was measured using the Higgins I^2^ statistic and Cochrane’s Q test (*P* < 0.10 or I^2^ > 50% was considered indicative of statistically significant heterogeneity) [17]. A random effects model (Der Simonian and Laird method) was applied if heterogeneity was present. However, the fixed-effect model was used in the absence of between-study heterogeneity (*P* > 0.10 or I^2^ < 50%). We performed subgroup analyses according to different subtypes of TILs as a sensitivity analysis to confirm the robustness of our results. Funnel plots were drafted for each meta-analysis to assess the potential publication bias.

## Results

### Search results and study characteristics

**Fig. 1:**
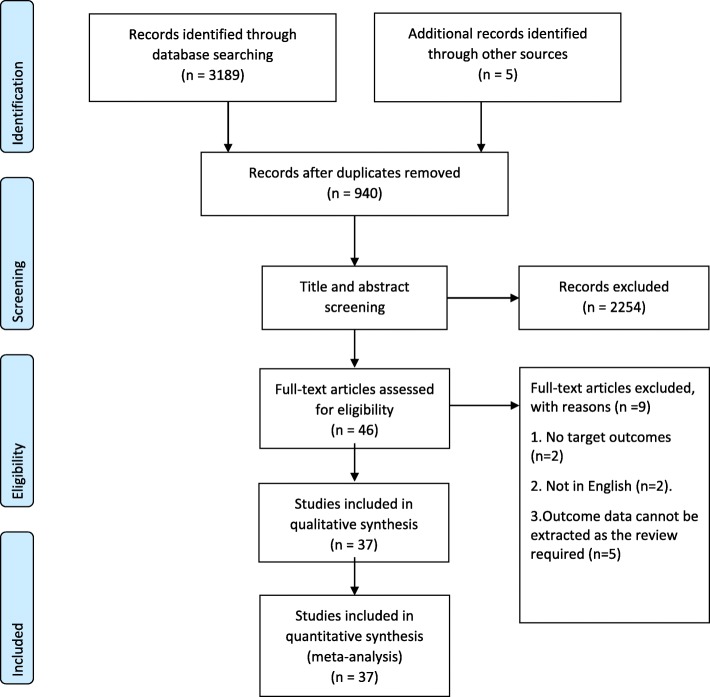
PRISMA flow diagram detailing the search strategy and results [11]

A total of 3194 articles were selected through searching the chosen electronic databases, and an additional 5 records were identified by cross-checking the bibliographies of retrieved meta-analysis or relevant reviews. After exclusion of duplicates, we screened the titles and abstracts and identified 46 articles for full-text review. We eliminated 9 papers according to the inclusion/exclusion criteria. Ultimately, 37 papers were included in the final analysis (Fig. 1) [7, 18–53].

The basic characteristics and target outcomes extracted from the included studies are listed in Table 1. All included articles (*n* = 37) were full-reported retrospective cohort studies. The studies were conducted in the United States (18.9%, 7/37), Japan (16.2%, 6/37), South Korea (16.2%, 6/37), China (8.1%, 3/37), France (8.1%, 3/37), Italy (3.4%, 2/37), Singapore (3.4%, 2/37), Germany (5.4%, 2/37), Australia (2.7%, 1/37), Peru (2.7%, 1/37), Spain (2.7%, 1/37), Canada (2.7%, 1/37), Ireland (2.7%, 1/37), and Switzerland (2.7%, 1/37). The population targeted was patients with TNBC. Eleven studies (29.7%, 11/37) provided evidence of the prognostic value of TILs for short-term outcomes (pCR), and five (75.7%, 28/37) provided evidence of the prognostic values of TILs for long-term outcomes (OS and/or DFS). The details of data extraction are presented in Additional file 2.

**Table 1:** Clinical details of the included studies

Author, year of publication	Country	Type of TNBC	No. of participants	TIL detection method	Location of TILs	Definition of high TIL level	TIL phenotype	Chemotherapy	Median follow-up (m)	Short-term prognosis	Target long-term prognosis
Adams et al. 2014 [18]	USA	Operable TNBC	481	HE	Intra-epithelial and stromal	TILs involving 50% of either tumor stroma or cell nests	None specified	AT&AC	127	not specified	DFS OS
AiErken et al. 2017 [19]	China	TNBC	215	HIC	Total and stromal	TILs-low (range, 0 to 10%); TILs-moderate (range, 11 to 40%); TILs-Marked (range, 41 to 100%).	PD-L1	Anthracyclines or Anthracyclines + Taxino	67.7	not specified	DFS OS
Althobiti et al. 2018 [20]	USA	TNBC	230	HE	Average stromal	Quantity of TILs was evaluated as percentage of TILs present in the stroma	CD3+ CD8+ FOXP3+ CD20+ CD68+	Not specified	not specified	not specified	OS
Asano et al. 2018 [21]	Japan	TNBC	61	HE	Stromal	> 10% was considered positive for TILs	None specified	Neoadjuvant	40.8	pCR	DFS
Byun et al. 2018 [22]	South Korea	TNBC	109	IHC	not specified	TILs were divided into (≥33% vs. < 33%) PD-L1 expression was categorized into two groups according to the final scores: low expression (< 100) and high expression (≥100).	PD-L1+ TILs	Not specified	76	not specified	DFS OS
Cerbelli et al. 2017 [23]	Italy	TNBC received standard NACT	54	IHC and HE	Stromal	TILs were quantified as a percentage of the stromal area of the tumor and expressed as a continuous parameter.	PD-L1	4 cycles of doxorubicin + cyclophosphamide Q3W followed by 12 cycles of paclitaxel weekly	not specified	pCR	not specified
Denkert et al. 2015 [24]	Germany	TNBC	255	IHC and HE	Stromal	TILs involving 60% of either tumor stroma or cell nests	PD1 PDL1 CD8+ FOXP3	Not specified	not specified	pCR	not specified
Denkert et al. 2018 [25]	Germany	TNBC	906	HE	Stromal	Three predefined categories: low TILs (0–10%), intermediate TILs (11–59%), or high TILs (60–100%).	None specified	(4 cycles of doxorubicin + cyclophosphamide Q3W followed by 12 cycles of paclitaxel weekly)	for OS, 62.8 months; median follow-up for DFS, 63.3 months	pCR	DFS OS
Dieci et al. 2014 [26]	France	TNBC patients with residual disease	293	HE	Intratumoral and stromal	High-TIL if It-TIL and/or Str-TIL > 60%	None specified	Neoadjuvant chemotherapy	75.6	not specified	OS
Dieci et al. 2015 [27]	France	TNBC	199	HE	Intratumoral and stromal	Cases were defined as High-TIL if It-TIL and/or Str-TIL > 60%	None specified	Not specified	152.4	not specified	OS
Galvez et al. 2018 [28]	Peru	TNBC	100	HE	Stromal	Cases were defined as High-TIL if Str-TIL > 50%	None specified	Neoadjuvant chemotherapy	not specified	pCR	not specified
Goto et al. 2018 [29]	Japan	TNBC treated with neoadjuvant chemotherapy	39	HE and IHC	Stromal	High if TILs occupied > 10% of the stromal area	CD8+ FOXP3^+^	standardised NAC protocol consisting of four courses of FEC100 (500 mg/m2 fluorouracil, 100 mg/m2 epirubicin and 500 mg/m2 cyclophosphamide) every 3 weeks, followed by 12 courses of 80 mg/m2 paclitaxel administered weekly.	not specified	not specified	OS
Herrero-Vicent et al. 2017 [30]	Spain	TNBC treated with neoadjuvant chemotherapy	164	HE	None specified	not specified	not specified	Standardised NAC protocol	not specified	pCR	not specified
Hida et al. 2016 [31]	Japan	TNBC	381	HE	None specified	Classified as high if TILs score > 50%	not specified	Neoadjuvant chemotherapy	45	pCR	not specified
Jang et al. 2018 [32]	South Korea	TNBC	231	HE	Stromal	Classified TILS as high (> 10%)	not specified	Anthracycline-based chemotherapy	117	not specified	DFS OS
Kim et al. 2017 [33]	South Korea	TNBC	40	HE	Stromal	Classified TILS score as high (> 60%).	Glutaminase^+^ TILs	An adjuvant methotrexate-based regimen	78.3	not specified	DFS
Krishnamurti et al. 2017 [34]	USA	TNBC without neoadjuvant treatments	157	HE	Stromal	TILs estimated in intervals as < 5, 5–10%, 11–50%, and > 50%	not specified	Not specified	not specified	not specified	DFS OS
Lee et al. 2016 [35]	South Korea	TNBC	769	HE	Stromal	TILs defined as the mean percentage of stroma of invasive carcinoma infiltrated by lymphocytes and plasma cells in 10% increments	not specified	Four cycles of adjuvant anthracycline and cyclophosphamide	not specified	not specified	DFS OS
Leon-Ferre et al. 2018 [36]	USA	TNBC	605	HE	Stromal and intratumoral	Lymphocyte-predominant breast cancer (LPBC) was defined as having > 50% stromal or intratumoral TILs	Not specified	Anthracycline and taxane	124.8	not specified	DFS OS
Li et al. 2016 [37]	USA	TNBC	136	IHC and HE	not specified	TILs were evaluated as the percentage of intratumoral stroma covered by mononuclear lymphocytes.	PD-L1 PD-1	Not specified	49.03	not specified	DFS OS
Loi et al. 2014 [38]	Australia	newly diagnosed TNBC	145	HE	Stromal	TILs ≥50%	not specified	Not specified	62	not specified	OS
Luen et al. 2019 [39]	France	TNBC treated with neoadjuvant chemotherapy	375	HE and IHC	Stromal	Quantification of TILs in the tumor stroma was recorded as a percentage of occupied stromal areas.	not specified	Anthracycline and taxane; Anthracycline alone; and Taxane alone	72	not specified	OS
Matsumoto et al. 2016 [40]	Singapore	Primary TNBC	232	HE and IHC	Stromal and intratumoral	Median TIL value as the cut-off for high vs. low	CD4^+^ CD8^+^	Not specified	not specified	not specified	DFS OS
McIntire et al. 2018 [41]	USA	TNBC	76	HE and IHC	None specified	TILs within the entire tumor were estimated at 5% intervals	CD8^+^	Not specified	110	not specified	DFS OS
Miyashita et al. 2014 [42]	Japan	TNBC	110	IHC	Stromal and intratumoral	None specified	CD8^+^ FOXP3+	Not specified	not specified	pCR	not specified
Mori et al. 2017 [43]	Japan	TNBC	248	IHC	Stromal and intratumoral	PD-L1+ was defined as expression in ≥5% of TILs	PD-L1	None specified	68	not specified	OS
O’Loughlin et al. 2018 [44]	Ireland	TNBC	75	HE	stromal	LPBC was defined as having > 50% stromal TILs	None specified	None specified	not specified	pCR	not specified
Ono et al. 2012 [45]	Japan	TNBC received NAC and subsequent surgical therapy	102	IHC	None specified	TIL score classified as high if the sum was 3–5	None specified	neoadjuvant anthracycline-based regimens	not specified	pCR	not specified
Park et al. 2016 [46]	South Korea	Early TNBC	133	HE	Stromal and intratumoral	Classified TILS as high (> 10%)	not specified	None specified	None specified	not specified	DFS OS
Pruneri et al. 2016 [47]	USA	TNBC	724	Multiplexed QIF staining	Stromal	LPBC defined as > 50% stromal TILs	not specified	Anthracycline + Taxanes ± CMF Anthracycline ± CMF	82.8	not specified	DFS OS
Pruneri et al. 2016 [48]	Switzerland	TNBC	897	HE	Stromal	None specified	not specified	CMF CMF + AC	98.4	not specified	DFS OS
Ruan et al. 2018 [49]	China	TNBC treated with neoadjuvant chemotherapy	166	None specified	Stromal and intratumoral	Classified TILS as high (> 10%)	not specified	Anthracycline + paclitaxel Paclitaxel + platinum	not specified	pCR	not specified
Seo et al. 2013 [7]	South Korea	TNBC	38	IHC	None specified	Median values of TILs used as cut off, and infiltration of TILs categorized as low or high.	CD4^+^ CD8^+^ FOXP3^+^	AC, AD; and ACT	not specified	pCR	not specified
Tian et al. 2016 [50]	China	Primary invasive TNBCs	425	HE	Stromal and intratumoral	LPBC was categorized as tumors involving ≥50% lymphocytic infiltration in either tumor stroma or cell nests	not specified	Anthracyclines; Anthracyclines + Taxanes	48	not specified	DFS OS
Urru et al. 2018 [51]	Italy	TNBC	841	IHC	Stromal	None specified	not specified	Not specified	51.6	not specified	DFS OS
West et al. 2013 [52]	Canada	TNBC	82	IHC	Stromal	None specified	FOXP3^+^ TILs	Not specified	not specified	not specified	DFS
Yeong et al. 2017 [53]	Singapore	TNBC	164	IHC	None specified	Cut-off median percentages used were also compatible to the accepted clinical pathological practices	FOXP3^+^	Not specified	not specified	not specified	DFS OS

*Abbreviations*: *TNBC* Triple negative breast cancer, *HE* Hematoxylin-eosin, *TNP* Triple-negative phenotype, *AC* Doxorubicin plus cyclophosphamide, *AT* Doxorubicin plus paclitaxel, *DFS* Disease-free survival, *OS* Overall survival, *IHC* Immunohistochemistry, *pCR* Pathological complete response, *LPBC* Lymphocyte-predominant breast cancer, *CMF* Cyclophosphamide methotrexate fluorouracil, *ACT* Doxorubicin plus cyclophosphamide followed by docetaxel, *AD* Doxorubicin plus docetaxel

### TILs and pCR

**Fig. 2:**
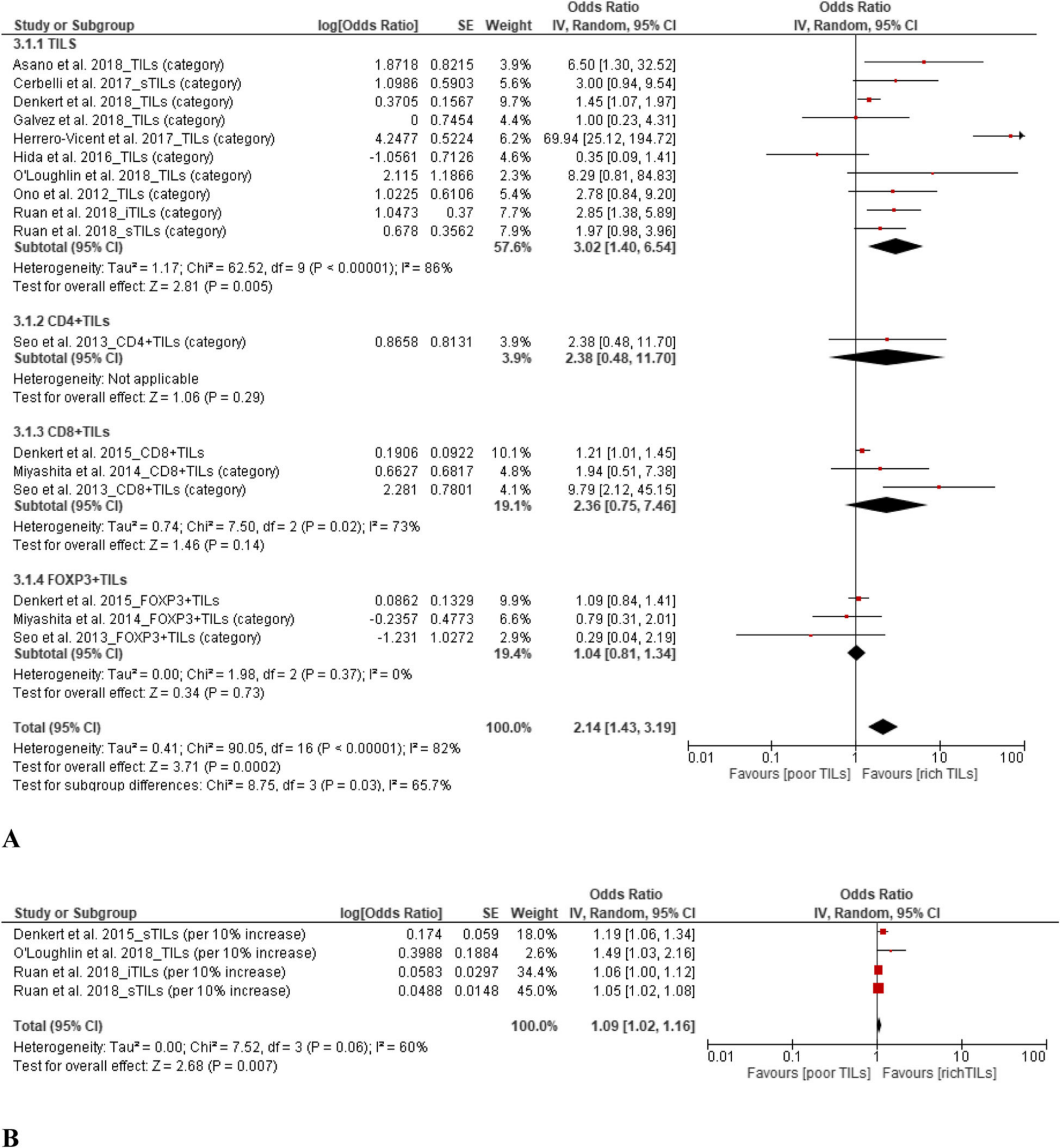
Forest plots of the random-effects meta-analysis for the efficacy of tumor-infiltrating lymphocytes (TILs) for predicting pathological complete response (pCR). **a** Low TILs vs. high TILs stratified by TIL phenotype. **b** Continuous TILs (10% increase) for pCR

From the 11 studies demonstrating the prognostic value of TILs for pCR among TNBC patients, the results showed that upregulation of TILs predicted a higher pCR rate. The pooled ORs were 2.14 (95% CI, 1.43–3.19) for TIL level (high vs. low) and 1.09 (95% CI, 1.02–1.16) for continuous TILs (10% increase in TIL level). When stratified by the TIL phenotypes of CD4^+^, CD8^+^, and FOXP3^+^, no statistical differences in pCR were found in the subgroup analysis. The details pooled results are presented in Fig. 2.

### TILs and OS

**Fig. 3:**
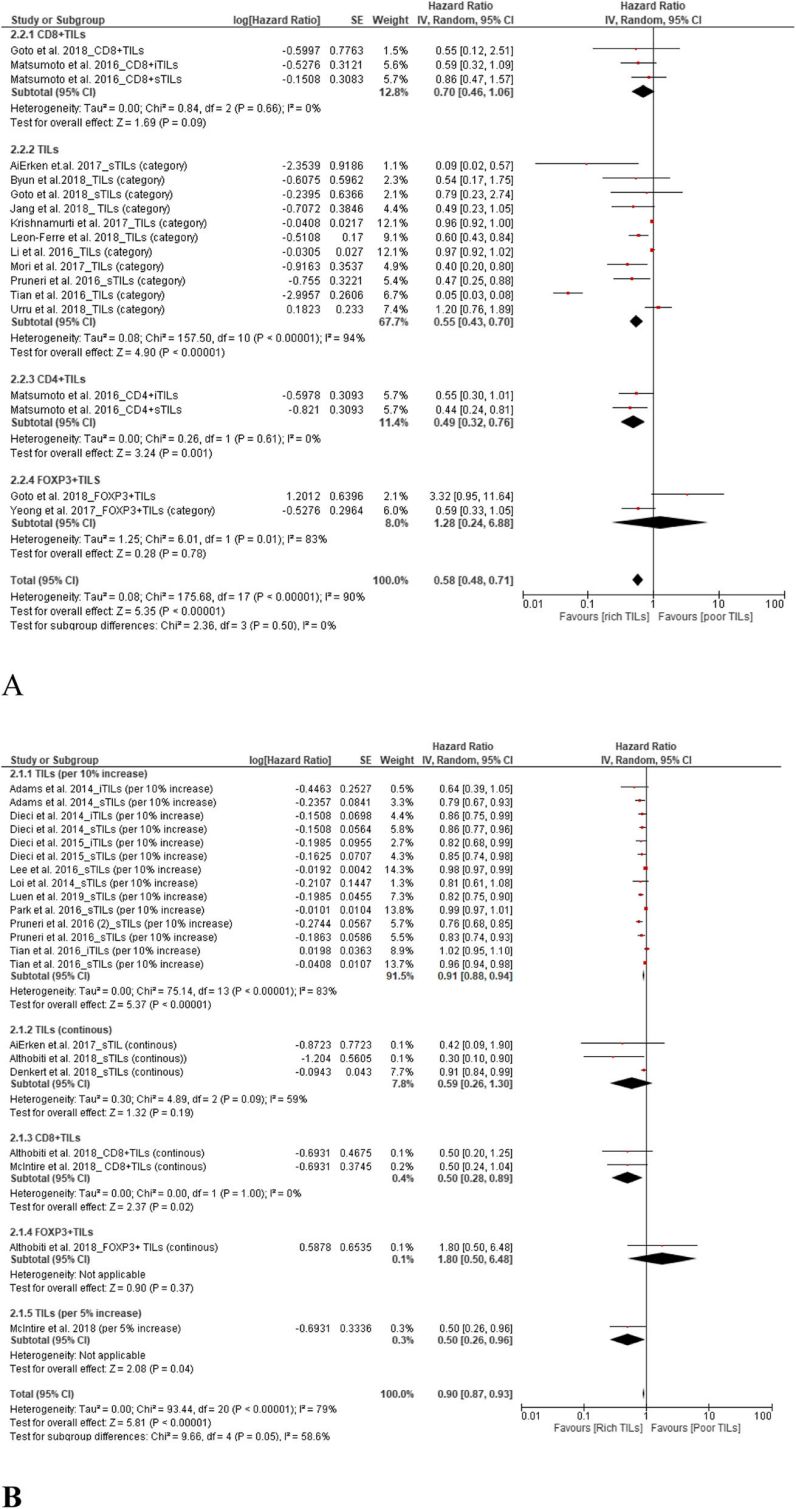
Forest plots of the random-effects meta-analysis for the efficacy of tumor-infiltrating lymphocytes (TILs) for overall survival (OS). **a** Low TILs vs. high TILs stratified by TIL phenotypes. **b** TILs stratified by continuous TILs, 5% increase in TILs, 10% increase in TILs, and phenotypes

A total of 24 studies supported the prognostic value of TILs for OS in TNBC patients. The results showed upregulation of TILs predicted a better OS. The pooled HRs were 0.58 (95% CI, 0.48–0.71) for total TIL level (high vs. low) and 0.90 (95% CI, 0.87–0.93) for continuous TILs (Fig. 3).

From subgroup analyses according to TIL phenotype (high vs. low), the HRs were 0.49 (95% CI, 0.32–0.76), 0.70 (95% CI, 0.46–1.06), and 1.28 (95% CI, 0.24–6.88) for CD4^+^ TILs, CD8^+^ TILs, and FOXP3^+^ TILs, respectively (Fig. 3a). Subgroup analyses according to the change in TIL level (continuous) returned HRs of 0.50 (95% CI, 0.28–0.89) and 1.80 (95% CI, 0.50–6.48) for CD8^+^ TILs and FOXP3^+^ TILs, respectively (Fig. 3b).

### TILs and DFS

**Fig. 4:**
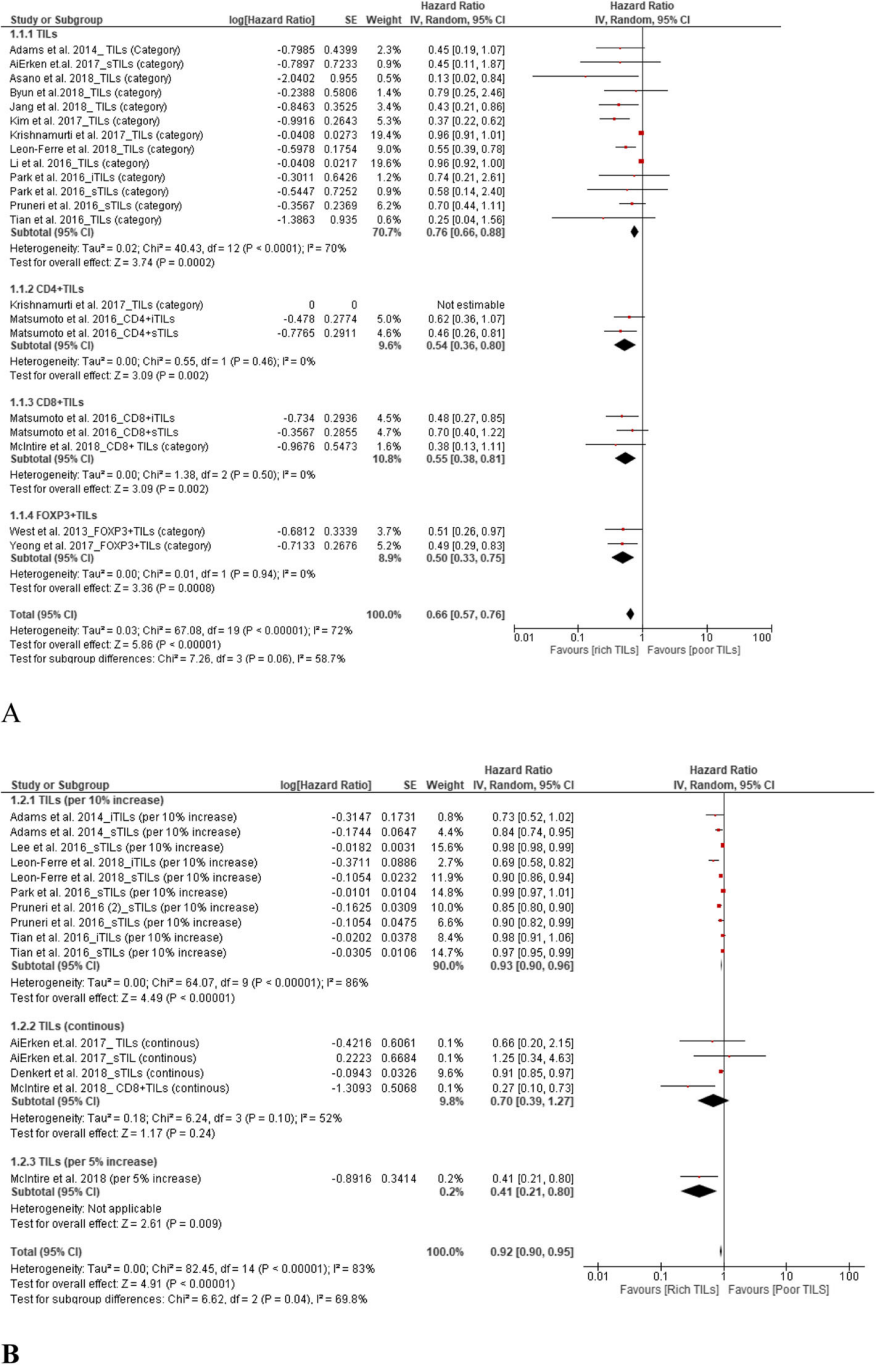
Forest plots of the random-effects meta-analysis for the efficacy of tumor-infiltrating lymphocytes (TILs) for disease-free survival (DFS). **a** Low TILs vs. high TILs stratified by TIL phenotype. **b** TILs stratified by continuous TILs, 5% increase in TILs, and 10% increase in TILs

A total of 20 studies supported the prognostic value of TILs for DFS in TNBC patients. The results showed upregulation of TILs predicted better DFS, with pooled HRs of 0.66 (95% CI, 0.57–0.76) for TIL level (high vs. low) and 0.92 (95% CI, 0.90–0.95) for continuous TILs (Fig. 4).

From subgroup analyses according to TIL phenotype (high vs. low), the HRs were 0.54 (95% CI, 0.36–0.80), 0.55 (95% CI, 0.38–0.81), and 0.50 (95% CI, 0.33–0.75) for CD4^+^ TILs, CD8^+^ TILs, and FOXP3^+^ TILs, respectively (Fig. 4a).

Subgroup analyses according to the change in TIL level (continuous) returned HRs of 0.93 (95% CI, 0.90–0.96), 0.70 (95% CI, 0.39–1.27), and 0.41 (95% CI, 0.21–0.80) for a 10% increase in TILs, continuous TILs, and a 5% increase in TILs of each subgroup, respectively (Fig. 4b).

### Risk of bias in included studies

**Fig. 5:**
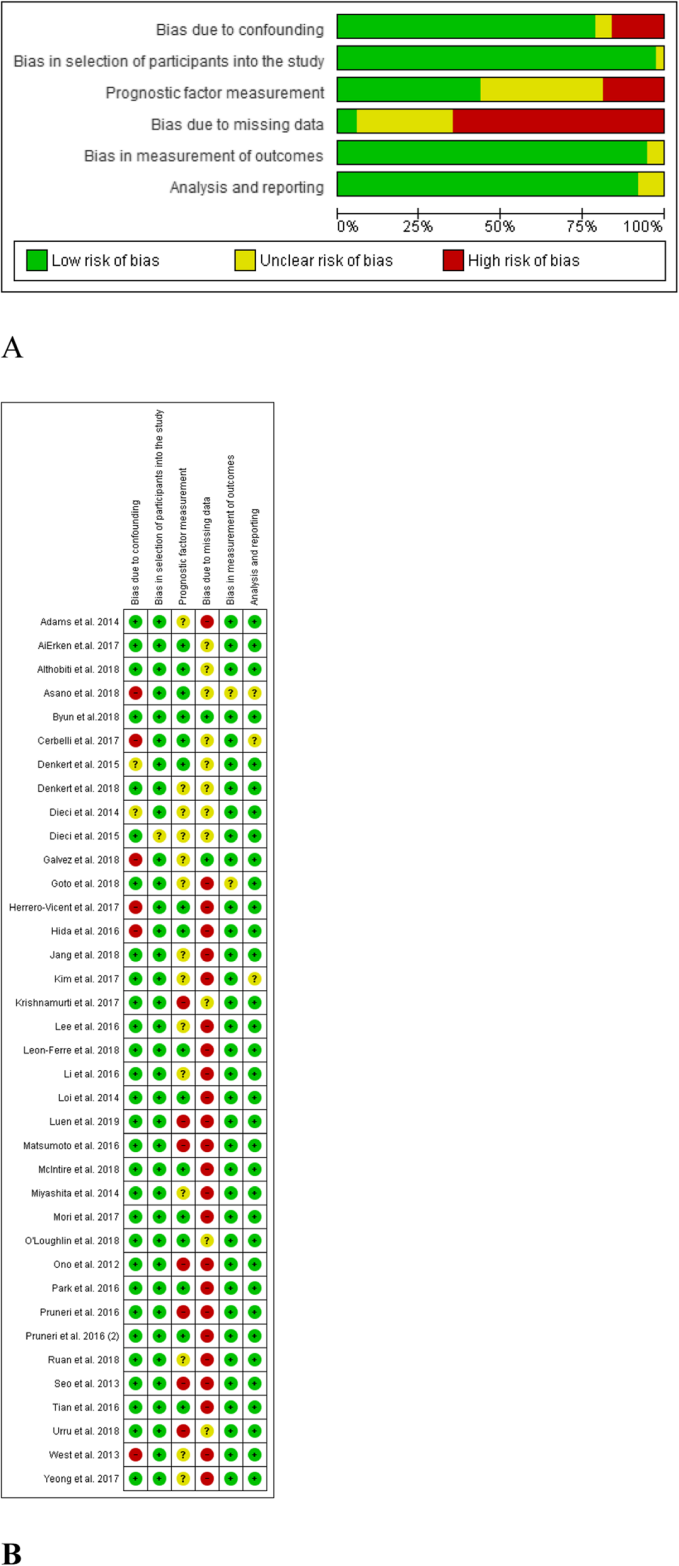
Risk of bias assessment at the study level. **a** Risk of bias graph: review authors’ judgements about each risk of bias item presented as percentages across all included full reported studies (*n* = 37). **b** Risk of bias summary: review authors’ judgements about each risk of bias item for each included study

We evaluated the risk of bias for all included studies (*n* = 37). We found the main sources of bias were related to missing data, TIL measurement and confounding controls. Most of the missing data due to that not all the available patients were included in the final analysis as the information was not complete (participants were excluded due to missing data). Figure 5a shows the risk of bias assessments for each cohort. Evaluations for each domain across full reported studies are shown in Fig. 5b.

### Publication bias

**Fig. 6:**
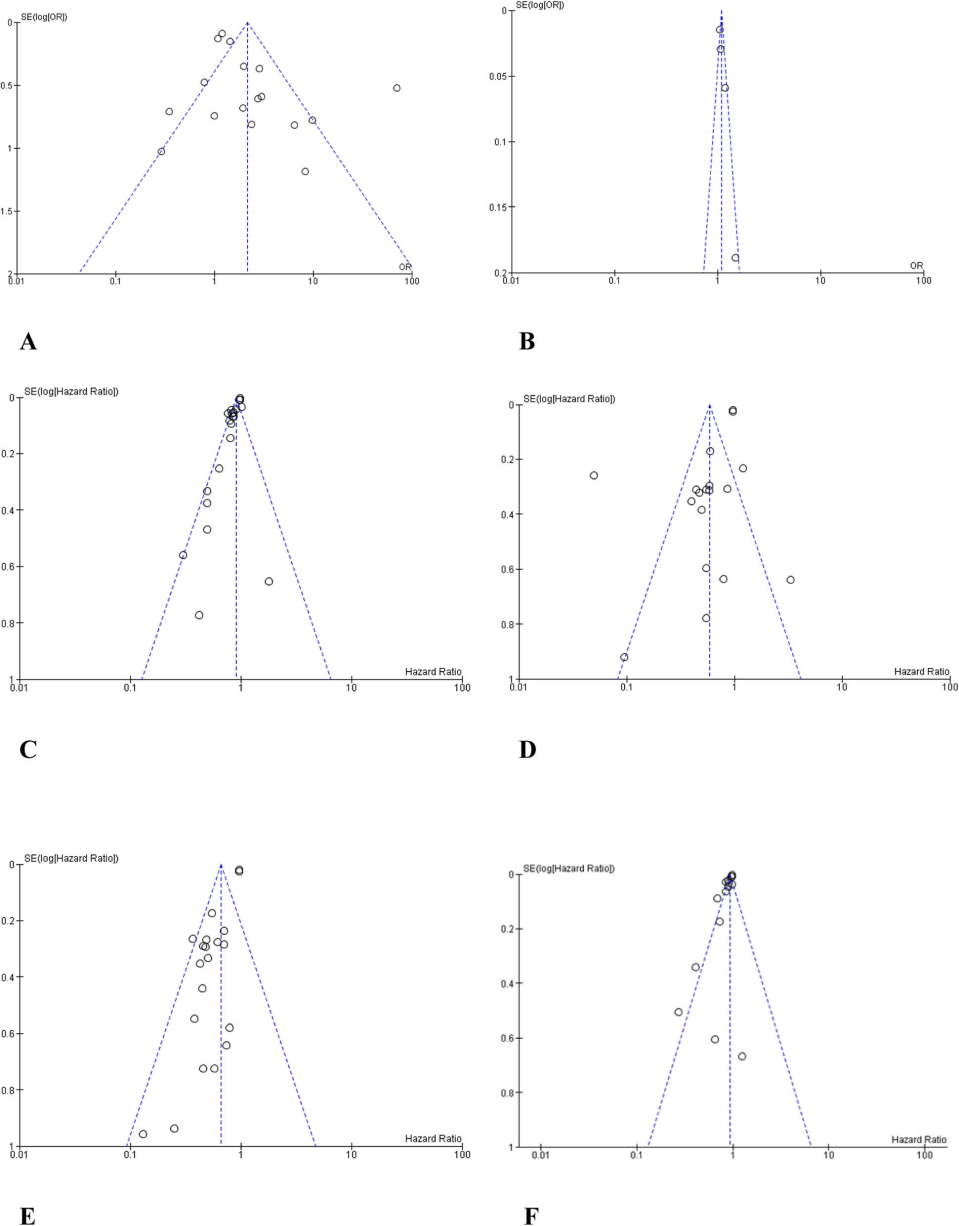
Funnel plot analysis of potential publication bias. **a** High tumor-infiltrating lymphocytes (TILs) vs. low TILs for pathological complete response (pCR). **b** Continuous TILs (10% increase) for pCR. **c** High TILs vs. low TILs for overall survival (OS). **d** Continuous TILs for OS. **e** High TILs vs. low TILs for disease-free survival (DFS). **f** Continuous TILs for DFS

Funnel plot analysis did not indicate apparent publication bias affecting the HRs for DFS and OS or the ORs for pCR in the included studies (Fig. 6).

## Discussion

As TNBC is a poor prognostic subtype of breast cancer, it is important to identify biomarkers that can rigorously predict its prognosis. The present review and meta-analysis synthesized 37 studies to evaluate the association between TIL levels, both total and specific subtypes, and prognosis in TNBC patients. Our findings indicate that a high TIL level in TNBC significantly increases the likelihood of pCR and improves DFS and OS.

In the present study, we used pCR as the indicator of short-term prognosis for patients with TNBC. Previous studies reported that higher TIL levels predict a better response to chemotherapy in patients with breast cancer [54–56]. According to our pooled results, compared to TNBC patients with low TIL levels, TNBC patients with high TIL levels had a higher rate of pCR to treatment (OR 2.14, 95% CI 1.43–3.19). Moreover, with each 10% increase in TIL level, patients with TNBC had an increased pCR rate (OR 1.09, 95% CI 1.02–1.16). A potential explanation for these findings is the influence of TILs to tumor immunosurveillance and tumor immunosuppression [57]. In addition, the treatment used in the included articles was inconsistent. However, no significant pCR improvement was observed for high levels of the CD4^+^, CD8^+^, and FOXP3^+^ TIL subgroups. This may due to the limited amount of data available for these subgroups.

The indicators of long-term prognosis in this study were OS and DFS. According to our pooled results, compared to TNBC patients with low TIL levels, patients with high TIL levels showed better OS (HR 0.58, 95% CI 0.48–0.71) and DFS (HR 0.66, 95% CI 0.57–0.76). Additionally, with a continuously increasing TIL levels, patients with TNBC had improved OS (HR 0.90, 95% CI 0.87–0.93) and DFS (HR 0.92, 95% CI 0.90–0.95). This finding is consistent with previous conclusions [3, 9, 25, 58, 59]. Our results indicate that a high level of TILs is a positive predictor for the prognosis of patients with TNBC.

The CD4^+^ TIL subgroup (high vs. low) showed a better OS (HR 0.49, 95%CI 0.32–0.76) and DFS (HR 0.54, 95%CI 0.36–0.80), and the CD8^+^ TIL subgroup (high vs. low) showed a better DFS only (HR 0.55, 95% CI 0.38–0.81). Nevertheless, the pooled results indicated CD4^+^ TILs and CD8^+^ TILs were positive predictors for long-term prognosis in TNBC. This is consistent with previous meta-analysis results [6]. The FOXP3^+^ TIL subgroup (high vs. low) also showed only better DFS (HR 0.50, 95% CI 0.33–0.75), with no statistical association with OS (HR 1.28, 95% CI 0.24–6.88). This finding for FOXP3^+^ TILs is opposite to that of previous meta-analyses [3, 6], and the reason for this inconsistency is unclear. More studies of the association of FOXP3^+^ TILs with the prognosis of TNBC are needed.

To our best knowledge, this was the first meta-analysis to pool the prognostic results for categorical TIL level and continuous TILs separately. Therefore, from the results, we can definitively conclude that a higher density of TILs corresponds to a better prognosis for TNBC. Our study does have some limitations. First, all included studies were retrospective cohort studies, with risks of bias related to missing data, TIL measurement, and confounding controls. Next, the variation in the definition of high/low TIL level, and the timeline(s) used for PFS and OS among the included studies can affect the accuracy of the results.

## Conclusions

TNBC with higher levels of TILs showed better short-term and long-term prognoses. High levels of specific phenotypes of TILs (CD4^+^, CD8^+^, and FOXP3^+^) could positively predict the long-term prognosis for TNBC.

## Supplementary information

**Additional file 1.** Literature search strategy. For additional file1, the content is the search strategies of EMBASE and MEDLINE. For additional file1, the content is the data extraction details of all included articles.

**Additional file 2.** The data extraction details for the included articles.

## Data Availability

Not applicable.

## Abbreviations

CD: Cluster of differentiation
CI: Confidence intervals
DFS: Disease-free survival
ER: Estrogen receptor
FBP3: Forkhead box P3
HER2: Human epidermal growth factor receptor 2
HR: Hazard ratios
IL: Interleukin
OR: Odds ratio
OS: Overall survival
pCR: Pathological complete response
PR: Progesterone receptor
PRISMA: Systematic Reviews and Meta-Analyses
QUIPS: Quality in Prognosis Studies
ROBIN I: Risk Of Bias In Non-randomised Studies - of Interventions
TILs: Tumor-infiltrating lymphocytes
TNBC: Triple-negative breast cancer
