# Supplementary material for: Prognostic value of tumor-infiltrating lymphocytes in patients with triple-negative breast cancer: a systematic review and meta-analysis
Source: BMC Cancer. 2020 Mar 4;20:179. doi: 10.1186/s12885-020-6668-z (PMC7057662; doi:10.1186/s12885-020-6668-z)
Supplement: Supplementary file 1 — Additional file 1. Literature search strategy. For additional file1, the content is the search strategies of EMBASE and MEDLINE. For additional file1, the content is the data extraction details of all included articles. [file 12885_2020_6668_MOESM1_ESM.docx]

**Appendix I. Literature search strategy.**

1. Database: OVID Medline Epub Ahead of Print, In-Process & Other Non-Indexed Citations, Ovid MEDLINE(R) Daily and Ovid MEDLINE(R) 1946 to Present

Search Strategy:

--------------------------------------------------------------------------------

1 (breast and (cancer or tumor or carcinoma or neoplasm) and ("triple negative" or "basal" or "basal-like") and (infiltrate or inflammatory or "immune response" or lymphocyte or "B cell" or "T cell" or CD4 or CD8 or CD40 or CD80 or "forkhead box transcription factor" or FOXP3) and (prognosis or prediction or predict or predicts or survival or mortality)).mp. [mp=title, abstract, original title, name of substance word, subject heading word, floating sub-heading word, keyword heading word, organism supplementary concept word, protocol supplementary concept word, rare disease supplementary concept word, unique identifier, synonyms] (594)

2 limit 1 to (english language and humans) (432)

***************************

2. Database: Embase <1974 to 2019 August 08>

Search Strategy:

--------------------------------------------------------------------------------

1 (breast and (cancer or tumor or carcinoma or neoplasm) and ("triple negative" or "basal" or "basal-like") and (infiltrate or inflammatory or "immune response" or lymphocyte or "B cell" or "T cell" or CD4 or CD8 or CD40 or CD80 or "forkhead box transcription factor" or FOXP3) and (prognosis or prediction or predict or predicts or survival or mortality)).mp. (2247)

2 limit 1 to (human and english language) (1936)

***************************

3. 823 results from Web of Science Core Collection between 1976 and 2019

You searched for: (TS=(breast and (cancer or tumor or carcinoma or neoplasm) and ("triple negative" or "basal" or "basal-like") and (infiltrate or inflammatory or "immune response" or lymphocyte or "B cell" or "T cell" or CD4 or CD8 or CD40 or CD80 or "forkhead box transcription factor" or FOXP3) and (prognosis or prediction or predict or predicts or survival or mortality))) AND LANGUAGE: (English) AND DOCUMENT TYPES: (Article)

Timespan: All years. Indexes: SCI-EXPANDED, SSCI, A&HCI, CPCI-S, CPCI-SSH, BKCI-S, BKCI-SSH, ESCI, CCR-EXPANDED, IC
