# Supplementary material for: Prognostic value of tumor-infiltrating lymphocytes in patients with triple-negative breast cancer: a systematic review and meta-analysis
Source: BMC Cancer. 2020 Mar 4;20:179. doi: 10.1186/s12885-020-6668-z (PMC7057662; doi:10.1186/s12885-020-6668-z)
Supplement: Supplementary file 2 — Additional file 2. The data extraction details for the included articles. [file 12885_2020_6668_MOESM2_ESM.docx]

Appendix II. The data extraction details for the included articles.

|  | Category of TILs | pCR | DFS (HR, 95% CI) | OS (HR, 95% CI) |
| --- | --- | --- | --- | --- |
| Adams *et al.* 2014 | Total (rich vs. poor) |  | 0.45 (0.19–1.03), P=0.06 |  |
|  | Intratumoral TILs (10% increase) |  | 0.73 (0.52–1.02), P=0.07 | 0.64 (0.39–1.05), P=0.08 |
|  | Stromal TILs (10% increase) |  | 0.84 (0.74–0.95), P=0.005 | 0.79 (0.67–0.92), P=0.003 |
| AiErken *et al.* 2017 | Stromal TILs (negative vs. positive) |  | 2.202 (0.535–9.057), P=0.274 | 10.549 (1.740–63.952), P=0.01 |
|  | Stromal TILs (Continuous TIL concentration) |  | 1.249 (0.337–4.632), P=0.74 | 0.418 (0.092–1.891), P=0.257 |
| Althobiti *et al.* 2018 | Stromal TILs (percentage) |  |  | 0.3 (0.1–0.8), P=0.023 |
|  | CD8^+^ TILs (percentage) |  |  | 0.5 (0.2–1.1), P=0.088 |
|  | FOXP3^+^ TILs (percentage) |  |  | 1.8 (0.5–6.1), P=0.313 |
| Asano *et al.* 2018 | Stromal TILs (positive vs. negative) | (26/48) vs. (2/13) | 0.13 (0.02–0.88), P=0.036 |  |
| Byun *et al.* 2018 | Total TILs (positive vs. negative) |  | 0.7876 (0.2524–2.4578), P=0.6810 | 0.5447 (0.1693–1.7525), P=0.3082 |
| Cerbelli *et al.* 2017 | Stromal TILs (positive vs. negative) | (11/22) vs. (8/32) |  |  |
| Denkert *et al.* 2015 | Stromal TILs (10% increase) | 1.19 (1.06–1.33), P=0.004 |  |  |
|  | CD8^+^ | 1.21 (1.01–1.46), P=0.04 |  |  |
|  | FOXP3^+^ | 1.09 (0.84–1.42), NS |  |  |
| Denkert *et al.* 2018 | Stromal TILs (Continuous TIL concentration) | (80/260) vs. (253/646)  1.17 (1.11–1.24), P<0.0001 | 0.91 (0.85–0.97), P=0.0028 | 0.91 (0.85–0.99), P=0.020 |
| Dieci *et al.* 2014 | Stromal TILs (per 10% increase) |  |  | 0.86 (0.77–0.97), P=0.01 |
|  | Intratumoral TILs (per 10% increase) |  |  | 0.86 (0.75–0.99), P=0.03 |
| Dieci *et al.* 2015 | Stromal TILs (per 10% increase) |  |  | 0.85 (0.74–0.99), P =0.04 |
|  | Intratumoral TILs (per 10% increase) |  |  | 0.82 (0.68–0.99), P=0.04 |
| Galvez *et al.* 2018 | Stromal TILs (positive vs. negative) | (6 /15) vs. (6 /15) |  |  |
| Goto *et al.* 2018 | Stromal TILs (negative vs. positive) |  |  | 1.270 (0.380–4.431), P=0.694 |
|  | CD8^+^ (low vs. high) |  |  | 1.822 (0.525–8.340), P=0.358 |
|  | FOXP3^+^ (high vs. low) |  |  | 3.324 (0.949–15.311),  P= 0.061 |
|  | CD8^+^/FOXP3^+^ (low) |  |  | 13.021 (2.241–258.136), P=0.002 |
| Herrero-Vicent *et al.* 2017 | sTILs (high vs. low) | (51/58) vs. (10/106) |  |  |
| Hida *et al.* 2016 | TILs (high vs. low) | (14/37) vs. (7/11) |  |  |
| Jang *et al.* 2018 | TILs (high vs. low) |  | 0.429 (0.215–0.859), P= 0.017 | 0.493 (0.232–1.047), P= 0.066 |
| Kim *et al.* 2017 | TILs (high vs. low) |  | 0.371 (0.221–0.623), P <0.001 |  |
| Krishnamurti *et al.* 2017 | Total TILs |  | 0.96 (0.91–1.01), P=0.11 | 0.96 (0.92–1.01), P=0.12 |
| Lee *et al.* 2016 | sTILs (10% increase) |  | 0.982 (0.976–0.989), P<0.001 | 0.981 (0.973–0.989), P<0.001 |
| Leon-Ferre *et al.* 2018 | sTILs (per 10% increment) |  | 0.90 (0.86–0.94), P=0.003 |  |
|  | iTILs (per 10% increment) |  | 0.69 (0.58–0.83), P<0.001 |  |
|  | TILs (high vs. low) |  | 0.55 (0.39–0.78), P<0.001 | 0.60 (0.43–0.83), P=0.001 |
| Li *et al.* 2016 | TILs (positive vs. negative) |  | 0.96 (0.92–1.00), P=0.0746 | 0.97 (0.92–1.01), P=0.1351 |
| Loi *et al.* 2014 | sTILs (per 10% increments) |  |  | 0.81 (0.61–1.1), P=0.14 |
| Luen *et al.* 2019 | sTILs (per 10% increment) |  |  | 0.82 (0.75–0.89), P<0.001 |
| Matsumoto *et al.* 2016 | CD8^+^ Stromal TILs (high vs. low) |  | 0.70 (0.40–1.21), P=0.1992 | 0.86 (0.47–1.57), P=0.6293 |
|  | CD8^+^ Intratumoral TILs (high vs. low) |  | 0.48 (0.27–0.83), P=0.0095 | 0.59 (0.32–1.07), P=0.0832 |
|  | CD4^+^ Stromal TILs (high vs. low) |  | 0.46 (0.26–0.82), P=0.0084 | 0.44 (0.24–0.83), P=0.0118 |
|  | CD4^+^ Intratumoral TILs (high vs. low) |  | 0.62 (0.36–1.07), P=0.0843 | 0.55 (0.30–1.01), P=0.054 |
| McIntire *et al.* 2018 | CD8^+^ TILs (low vs. high) |  | 2.63 (0.92–7.48), P=0.07 |  |
|  | TILs (per 5% increment) |  | 0.41 (0.21–0.70), P=0.003 | 0.50 (0.26–0.87), P=0.02 |
|  | CD8+ TILs (Continuous Variable) |  | 0.27 (0.10–0.71), P=0.008 | 0.50 (0.24–1.08, P=0.08 |
| Miyashita *et al.* 2014 | CD8^+^ TILs (high vs. low) |  | 1.94 (0.51–7.73), P=0.329 |  |
|  | FOXP3^+^ TILs (high vs. low) |  | 0.79 (0.31–2.04), P=0.632 |  |
|  | CD8^+^/FOXP3^+^ (high vs. low) |  | 5.32 (1.62–19.98), P=0.005 |  |
| Mori *et al.* 2017 | TILs (high vs. low) |  |  | 0.4 (0.2−0.8), P=0.015 |
| O'Loughlin *et al.* 2018 | sTILs (positive vs. negative) | 8.29 (0.81–84.55), P=0.074 |  |  |
|  | sTILs (10% increments) | 1.49 (1.03–2.15), P=0.035 |  |  |
| Ono *et al.* 2012 | TILs (high vs. low) | 2.78 (0.84–9.18), P=0.09 |  |  |
| Park *et al.* 2016 | sTILs (per 10% increase) |  | 0.99 (0.97–1.01), P=0.44 | 0.99 (0.97–1.02), P=0.94 |
|  | sTILs (positive vs. negative) |  | 0.58 (0.14–2.35), P=0.45 | 0.91 (0.20–4.08), P=0.91 |
|  | iTILs (positive vs. negative) |  | 0.74 (0.21–2.54), P=0.63 | 0.52 (0.12–2.17), P=0.37 |
| Pruneri *et al.* 2016 | sTILs (10% increment) |  | 0.9 (0.82–0.97), P=0.01 | 0.83 (0.74–0.93), P<0.001 |
|  | sTILs (positive vs. negative) |  | 0.7 (0.44–1.11), P=0.13 | 0.47 (0.25–0.89), P=0.02 |
| Pruneri *et al.* 2016 | sTILs (10% increment) |  | 0.85 (0.80–0.92), P<0.0001 | 0.76 (0.68–0.84), P<0.0001 |
| Ruan *et al.* 2018 | sTILs (10% increment) | 1.05 (1.02–1.09), P=0.006 |  |  |
|  | iTILs (10% increment) | 1.06 (1.00–1.12), P=0.04 |  |  |
|  | sTILs (positive vs. negative) | 2.85 (1.38–5.90), P=0.005 |  |  |
|  | iTILs (positive vs. negative) | 1.97 (0.98–3.98), P=0.06 |  |  |
| Seo *et al.* 2013 | CD4^+^ TILs (negative vs. positive) | 2.377 (0.483–11.688), P=0.287 |  |  |
|  | CD8^+^ TILs (negative vs. positive) | 9.786 (2.121–45.149), P=0.003 |  |  |
|  | FOXP3^+^ TILs (negative vs. positive) | 0.292 (0.039–2.212), P=0.234 |  |  |
| Tian *et al.* 2016 | sTILs (per 10% increase) |  | 0.97 (0.95–0.99), P=0.001 | 0.96 (0.94–0.99), P=0.003 |
|  | iTILs (per 10% increase) |  | 0.98 (0.91–1.05), P=0.53 | 1.02 (0.95–1.10), P=0.61 |
|  | TILs (positive vs. negative) |  | 0.25 (0.04–1.83), P=0.17 | 0.05 (0.03–13.6), P=0.29 |
| Urru *et al.* 2018 | TILs (positive vs. negative) |  |  | 1.20 (0.76–1.91) |
| West *et al.* 2013 | FOXP3^+^ TILs (positive vs. negative) |  | 0.506 (0.263–0.972), P=0.041 |  |
| Yeong *et al.* 2017 | FOXP3^+^ (High vs. low) |  | 0.49 (0.29–0.83), P=0.008 | 0.59 (0.33–1.04), P=0.068 |

Abbreviations: DFS, disease-free survival; OS, overall survival; TILs, tumor infiltrating lymphocytes; pCR, pathologic complete response; PD-L1, Programmed death ligand 1; and sTILs, stromal lymphocytic infiltrate
